# Mr. Lowe, on Hydrophobia

**Published:** 1804-11-01

**Authors:** Robert Lowe

**Affiliations:** Member of the Royal College of Surgeons, London. Tavistock Row, Covent Garden


### *To the Editors of the Medical and Physical Journal.*

Gentlemen,

NO errors should be permitted to pass unnoticed in a work so widely diffused as your Medical Journal, which contains a fund of useful documents, that are not only consulted with pleasure and instruction by the experienced practitioner, but which are also greedily sought after for information by the juvenile tyro, whose mind has not been sufficiently expanded over the wide ocean of medical science, to enable him to shun those dangerous rocks with which it abounds, or to avoid his being mislead by those *wild* and *extravagant fancies*, which speculators in physic are for ever throwing in his way.

Your correspondent, Mr. Hume, (whose observations on the nomenclature of modern pharmacopœias, in a former number of your Journal, are very worthy of notice) I now perceive begins to turn his mind to surgery; and having taken the hint from Mr. Hardman, in your last number, proposes the application of *exhausted cupping glasses* as a preventive and *immediate cure* of hydrophobia.

Fortunately for mankind, the hydrophobic fever, or mania, does not occur sufficiently often to allow of many experiments being made, and a plan of treatment has been long known and practised with the greatest success in preventing its occurrence; but I am sorry to say, that very little progress has been made beyond this, for we are completely ignorant of any very successful mode of treating it, when its symptoms do occur.

The

The application of suction to wounds, which have been inflicted by *rabid* or *venomous animals,* was well known to and extolled by the antients, and I believe it is practised to this day, in those barbarous countries where poisons are used to render more dire and destructive their implements of carnage.

Suction, if likely to be of any kind of service in preventing hydrophobia, ought to be applied immediately after the injury has been received; it operates by taking off atmospherical pressure, and promoting a discharge of fluids from the wounded parts, if recent. We are now to suppose, that, with these fluids, the whole of the poison which has been inserted by the *rabid animal,* will be extracted from the wounds, otherwise we cannot expect that our operation will be productive of the intended effect. Now I will candidly ask Mr. Hume, if he suppose such an effect certain, or very likely to be produced, or that such a practice ought to be depended upon?

But to examine the matter a little further; Mr. Hume not only recommends the application of *cupping glasses* as a preventive, but as an *immediate cure* of hydrophobia: I think that I have already proved in the most satisfactory manner, how little dependance should be placed on such an application as a preventive of *hydrophobia*; nor will it be very difficult for me to show, that when the *disease* has actually taken place, the application of *cupping glasses* will be of no service whatever.

When the disease termed hydrophobia has taken place, which may be known by the great *anxiety, fever,* and *dread of* swallowing fluids, it is supposed, from analogy, supported by a great many facts, that *matter* of a peculiar nature, which had been lodged in the wounds inflicted by the *rabid animal,* is become absorbed into the system, and has produced its specific effect. Presuming this to be the case, what advantage is likely to be derived from the application of *cupping glasses?* One might as well apply *cupping glasses* over the arm of a child inoculated for the small-pox, with an intention of checking or renewing the eruption and fever when they have made their appearance. The application of powerful *caustics,* or excision of the immediately contiguous parts, I believe, are the only remedies to be depended on, in the prevention of *hydrophobia*; nor is there, to my knowledge, any case on record of their having failed of producing that effect, when extensively and properly applied; and in a case which

 came came under my care upwards of three years ago, I applied caustic with success, notwithstanding my patient, for a long time, laboured under the most dreadful apprehensions for the consequences; the animal (pointer dog) too, in this case, exhibited the most unequivocal symptoms of the *disease*.

The best kind of caustic, and what I employed in the above case, is the *calx cum kali puro*, commonly termed lapis infernalis.

Most of the animal poisons have their effects upon the system weakened by the internal and external exhibition of alkalis; if this be the case with the *poisonous matter* producing *hydrophobia*, we might be led to expect a double effect from the alkali contained in the above caustic.

I cannot close my remarks without stating a surprising fact, well known to many practitioners, viz. that it is not uncommon for the wounds inflicted by a *mad animal*, to heal as kindly as similar wounds from an animal in health; yet the disease at length has been produced, sometimes after a lapse of several months from the accident: this shows us how fallacious the rule is, which I know to have been practised, of determining upon the nature of the case from its rapid or tardy progress towards healing; it also gives us confidence in a practice, which may be successful in preventing the disease a long time after the accident has been received.

I am, &c.

*Tavistock Row, Covent Garden, Oct.* 7, 1804. ROBERT LOWE,

Member of the Royal College of Surgeons, London.

